# Health outcomes of umbilical cord clamping techniques in preterm neonates: meta-analysis

**DOI:** 10.1590/1806-9282.20241480

**Published:** 2025-07-04

**Authors:** Fatma Şule Bilgiç, Aysu Yıldız Karaahmet, Altan Alaybeyoğlu

**Affiliations:** 1 Çanakkale Onsekiz Mart University, Faculty of Health Science, Department of Midwifery – Çanakkale, Turkey.; 2 Biruni University, Faculty of Health Science, Department of Midwifery – İstanbul, Turkey.; 3 Haliç University, Faculty of Business Administration, Department of Management Information Systems – İstanbul, Turkey.

**Keywords:** Prematurity, Umbilical cord milking, Cord clamping, Hemoglobin, Hematocrit, Ferritin, Bilirubin

## Abstract

**OBJECTIVE:**

The aim of this study was to determine the effect of umbilical cord clamping time and milking on blood parameters in preterm neonates.

**METHODS:**

A literature search was conducted between July and September 2024 in four databases. The search was performed using MeSH-based keywords.

**RESULTS:**

In this study, the results of 14 studies covering a total of 1,609 preterm neonates were analyzed. Follow-up after intervention showed no statistically significant difference in hemoglobin (standardized mean difference=0.22, 95%CI 0.04–0.48, Z=1.65, p=0.10) and bilirubin (standardized mean difference=0.22, 95%CI 0.30–0.74, Z=0.82, p=0.41) between the groups. There was a statistically significant difference in ferritin (standardized mean difference=0.73, 95%CI 0.30–1.15, Z=3.37, p=0.00008) and hematocrit (standardized mean difference=0.30, 95%CI 0.05–0.54, Z=2.41, p=0.02) values, and the effect size was positive. According to the subgroup analysis of the combined results of the studies, it was seen that there was no statistically significant difference in the adverse health outcomes in preterms (OR 0.92, 95%CI 0.74–1.15, Z=0.72, p=0.47).

**CONCLUSION:**

From the analysis, it can be observed that late cord clamping and cord milking can prevent premature anemia by increasing hematocrit and ferritin formation in preterm newborns.

## INTRODUCTION

Babies born before completing the 37th week of pregnancy in the womb are considered premature^1^. These babies have low hemoglobin (Hb) values, which is defined as premature anemia. Anemia of prematurity has been associated with low Hb values at birth for gestational age, short erythrocyte lifespan, low endogenous erythropoietin, hyporegenerative bone marrow, medical complications, iatrogenic blood loss, and rapid growth^2,3^.

The two most common interventions to ensure placental perfusion at birth are milking and late clamping of the umbilical cord. Delaying cord clamping for at least 30–60 s in term infants is beneficial in terms of keeping Hb levels and iron stores high in the first months, and iron deficiency anemia is less common in infants whose cords are clamped late. In preterm infants, late cord clamping can increase erythrocytes, reduce transfusion requirements and the incidence of necrotizing enterocolitis, and reduce the development of intraventricular hemorrhage (IVH). Therefore, in the ACOG recommendations updated in 2020, it is advised to wait at least 30–60 s after birth for term and healthy preterm infants and clamp the cord. No side effects were observed with late clamping in term and preterm infants other than polycythemia and non-serious hyperbilirubinemia. Although the ideal cord clamping time in premature babies is not clearly known, early clamping (<30 s) is not recommended^2-7^.

Cord milking is performed by gently milking the umbilical cord 2–4 times at a constant speed while keeping it at a distance of 20–30 cm toward the newborn^5,6^. It has been stated that umbilical cord milking (UCM) increases the risk of IVH and respiratory distress syndrome in extremely premature infants and may have side effects due to polycythemia. The literature on the effect of umbilical cord interventions to reduce anemia, which continues to be a threat for premature infants, is still contradictory^8^. The aim of this study was to systematically review and meta-analyze the results regarding the effect of umbilical cord clamping time and milking on blood parameters in premature newborns.

## METHODS

### Eligibility criteria

The studies to be included in the study were decided by taking into account the PICOS criteria:

Participant (P): Preterm newborns: The criteria for the inclusion of preterm newborns in the study were as follows: (1) no chromosomal anomaly, (2) no congenital anomaly, (3) 37 gestational week (GW) small, (4) no placental anomaly, and (4) no Rh nonconformity.

Intervention (I): Cord clamping (1): UCM and (2): delayed cord clamping (DCC).

Comparison (C): (1) Immediate cord clamping (ICG) and (2) DCC.

Outcomes (O): (1): Hb, (2) hematocrit (3) peak bilirubin level, (4) serum ferritin, (5) IVH, (6) necrotizing enterocolitis, (7) anemia, (8) respiratory distress syndrome, (9) patent ductus arteriosus, and (10) neonatal polycythemia.

Study design (S): Randomized controlled trials (RCTs) were included.

### Search strategy

This systematic review and meta-analysis included full-text accessible RCTs published in academic journals. The literature review was conducted between July and September 2024 using four electronic databases (PubMed, CINAHL, Scopus, and ULAKBIM). For the literature review, first of all, the keywords in the Medical Subject Headings 2023 thesaurus were determined. The keywords were "preterm" AND "infant" OR "baby" OR" newborn" OR "delayed cord clamping" OR "Cord milking" OR "immediate cord clamping" OR "blood parameters" OR "hemoglobin" OR "hematocrit" OR "bilirubin" OR "ferritin."

### Data analysis

Review Manager 5.4.1 software was used to analyze the articles. For binary variables (effective rate), relative risk ratio (RR) and 95%CI were used as effect indicators. For continuous variables, weighted standardized mean difference (SMD) or mean difference (MD) with 95%CI was used as an effect indicator. Heterogeneity testing was performed using I^2^ value and Q test. When I^2^≥50%, some heterogeneity was considered, and a random-effects model was used, while when I^2^≤50%, the included articles were considered homogeneous and a fixed-effects model was used. The risk of bias for each area was classified as "low risk," "high risk," or "uncertain risk" according to the decision criteria in the "bias risk" assessment tool.

## RESULTS

### Literature review

#### Study characteristics

This systematic review and meta-analysis included 14 studies on preterm newborns, in which a total of 1,609 preterm newborns were included to review the results on the effect of umbilical cord clamping time and milking on blood parameters and to evaluate the available evidence. In all the studies included in the analysis, preterm newborns were treated with an intervention related to early or late cord clamping or cord milking^7,9-22^ (Table 1).

**Table 1:** Characteristics and main findings of the studies included in the systematic review and meta-analysis (n=14).

Reference\country	Population	Gestational age	Umbilical cord protocol	CG
Prachukthum et al.^18^, Thailand	80 PN: UCM: 40, CG: 40	28+0 to 33+6	UCM on about 25 cm length of the cord three times, then cutting the umbilical cord.	CG: DCC
Josephsen et al.^13^, USA	59 PN: UCM: 30, CG: 29	24+0/7 to 27+6/7	Cord milking was performed by milking 18 cm of the cord beginning at the placental end and continuing toward the infant three times.	CG: ICG
Sura et al.^20^, Kenya	280 PN: UCM: 140, CG: 140	28 to <37	The cord was milked four times with an interval of 2 s.	CG: DCC
Atia et al.^9^, Southern Region	200 PN: UCM: 100, CG: 100	24+0 to 34+6	The cord was milked four times with an interval of 2 s.	CG: DCC
Finn et al.^10^, Ireland	200 PN: UMC: 18, CG: 12	<32 weeks	The cord was milked three times at 2-s intervals from a distance of 20 cm.	CG: ICG
Knol et al.^16^, the Netherlands	39 PN: PBCC: 22, CG: 17	<32 weeks	The cord was not cut until the baby's vital signs were stable.	CG: DCC
Shirk et al.^19^, USA	204 PN: UCM: 104, CG: 100	23+0 to 34+6	The cord was milked three times at 2-s intervals from a distance of 20 cm.	CG: DCC
El-Naggar et al.^11^, Canada	73 PN: UCM: 37, CG: 36	24+0 to 30+6	The cord was milked three times at 2-s intervals from a distance of 20 cm.	CG: ICG
Ram Mohan et al.^22^, India	60 PN: UCM: 30, CG: 30	≤37 weeks	The cord was milked three times at 2-s intervals from a distance of 20 cm.	CG: ICG
Kilicdag et al.^15^, Turkey	54 PN: UCM: 29, CG: 25	≤32 weeks	The cord was milked three times at 2-s intervals from a distance of 20 cm.	CG: ICG
Kumar et al.^17^, India	200 PN: UCM: 100, CG: 100	32+0 to 36+6	The cord was milked three times at 2-s intervals from a distance of 25 cm.	CG: ICG
Ranjit et al.^21^, India	100 PN: DCC: 50, CG: 50	30+0 to 36+6	In the DCC group, the cord was clamped before 2 min of the delivery of the infant	CG: ICG
Elimian et al.^12^, California	200 PN: DCC: 99, CG: 101	24+0 to 34+0	Three to four passes of milking of the umbilical cord toward the neonate was allowed in all neonates in the DCC group.	CG: ICG
Katheria et al.^14^, California	60 PN: UCM: 30, CG: 30	<32 weeks	The cord was milked three times at 2-s intervals from a distance of 20 cm.	CG: ICG

PN: preterm neonates; UCM: umbilical cord milking; DCC: delayed cord clamping; ICG: immediate cord clamping; PBCC: physiological-based cord clamping; CG: control group..

#### Outcomes

Hb and hematocrit were assessed in all studies included in the analysis^7,9-22^. Six studies reported bilirubin levels and three studies reported ferritin value.

#### Blood parameters

**Figure 1:**
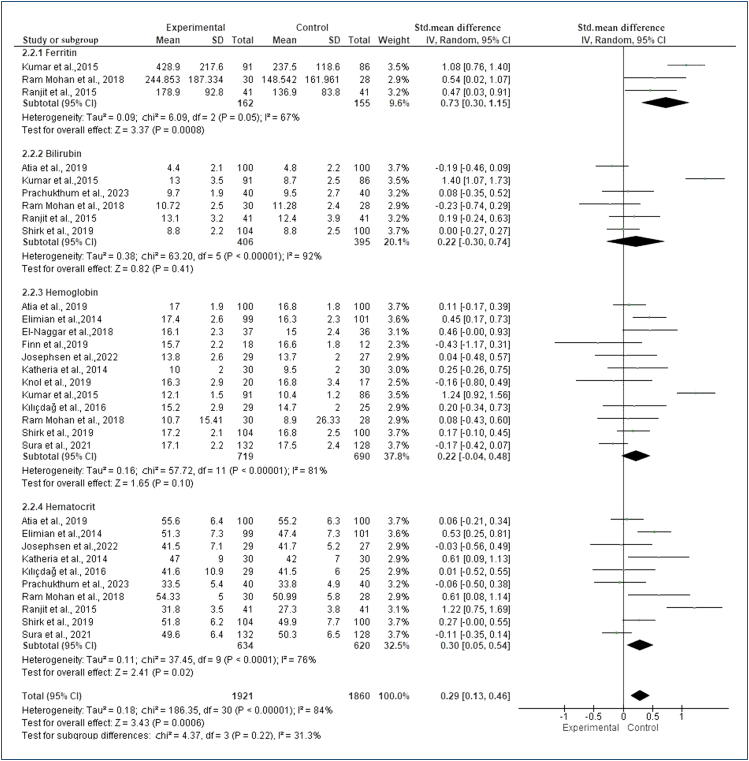
Forest plot for comparison: meta-analysis results on the effect of interventions on hematological parameters.

A total of 3 of the reviewed studies reported ferritin, 6 bilirubin, 12 Hb (g/dL), and 10 hematocrit results. When the mean pooled results of the studies were examined, a high level of heterogeneity was found (I^2^=84%, p<0.00001). There was a statistically significant difference in ferritin (SMD=0.73, 95%CI 0.30–1.15, Z=3.37, p=0.00008) and hematocrit (SMD=0.30, 95%CI 0.05–0.54, Z=2.41, p=0.02) values and the effect size was positive (Figure 1).

#### Preterm health outcomes

**Figure 2:**
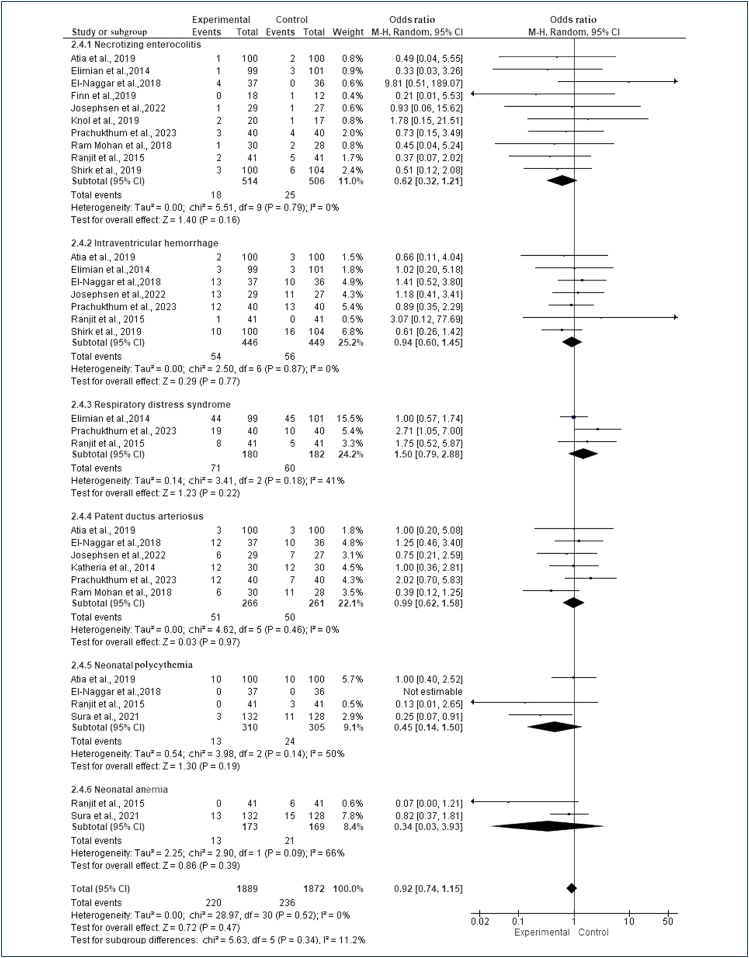
Forest plot of comparison: meta-analysis results on the effect of interventions on preterm health outcomes.

The results regarding the adverse health outcomes of necrotizing enterocolitis, IVH, respiratory distress syndrome, patent ductus arteriosus, neonatal polycythemia, and neonatal anemia are depicted in Figure 2. According to the subgroup analysis of the combined results of the studies, it was seen that there was no statistically significant difference in the adverse health outcomes in preterms (OR 0.92, 95%CI 0.74–1.15, Z=0.72, p=0.47; Figure 2).

#### Risk-of-bias assessment

In the 13 studies included in the analysis, it was seen that there was a sufficient method for randomly assigning preterm newborns to groups^7,9-14,16-22^. Therefore, the risk of side-keeping was evaluated as low in these studies. In the study by Kilicdag et al.^15^, it was determined that there was an unclear risk of favoritism since there was no explanation for randomization. In all studies, since it was assigned to groups using sequentially numbered and sealed opaque envelopes, bias assessed the risk as low. While blindness was not required in participants due to the nature of the study, the risk was low because 13 studies provided blindness in terms of statisticians and randomization.

## DISCUSSION

It is recommended not to perform early cord clamping in order to increase survival rates and reduce morbidity in preterm newborns^23^. The aim of this study was to determine the effect of umbilical cord clamping time and milking on blood parameters in preterm neonates (PNs). In this meta-analysis, late cord clamping and cord milking increased Hb, hematocrit, and ferritin levels in preterm infants. Additionally, there was no difference in the incidence of negative health outcomes compared to the control group.

A meta-analysis of 18 RCTs on early and late cord clamping reported that blood transfusion rates decreased by 10% and hematocrit increased by 2.7% with late cord clamping^2^. Another meta-analysis indicated that late cord clamping may improve hematological parameters in preterm infants; however, further studies on cord milking are needed. The ideal umbilical cord interventions for preterm infants are not yet clearly defined, but early clamping may be harmful. In the meta-analysis by Gomersall et al.^24^, it was found that late cord clamping reduced the need for blood transfusion and increased Hb levels in term and late preterm infants. Another meta-analysis reported that cord milking reduces the need for blood transfusion in PNs^25^. Furthermore, a meta-analysis conducted on term infants showed that cord milking improved hematological outcomes more than DCC^26^. In summary, both late cord clamping and cord milking may help prevent premature anemia by increasing hematocrit and ferritin levels in preterm infants. Regarding Hb levels, a positive effect size was observed, indicating that these interventions may increase Hb values.

A meta-analysis study on early and late cord clamping reported that in-hospital mortality decreased with late clamping, but no effects were found on IVH, chronic lung disease, necrotizing enterocolitis, or retinopathy^2^. Another meta-analysis found that the duration of cord clamping increased the risk of respiratory distress syndrome in term and late preterm infants^24^. Another meta-analysis reported that late cord clamping reduced the risk of mortality in preterm infants compared to ICG^27^. Additionally, a meta-analysis study indicated that cord milking increased the risk of intracranial hemorrhage, particularly in preterm infants born at earlier gestational ages, compared to DCC^25^. Another meta-analysis similarly reported that DCC reduced IVH in preterm infants compared with immediate clamping^28^. Although the findings in this study are consistent with the existing literature, further research is needed to better define the optimal interventions for PNs.

## CONCLUSION

From this analysis, it can be said that late cord clamping and UCM may prevent premature anemia by increasing hematocrit and ferritin formation in preterm newborns. Based on Hb results, it was found that late cord clamping and UCM may increase Hb values since the effect size was positive in addition to the power of causing negative health performance.
